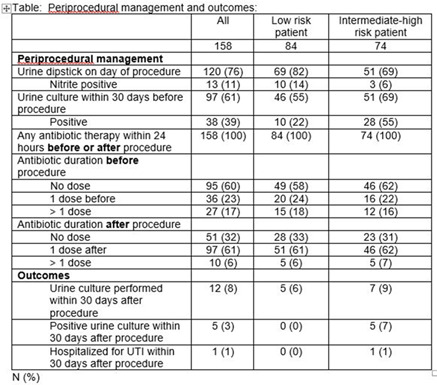# Periprocedural Testing and Antimicrobial Prophylaxis for Ureteral Stent Removal: An Opportunity for Antimicrobial Stewardship

**DOI:** 10.1017/ash.2021.52

**Published:** 2021-07-29

**Authors:** Dhatri Kotekal, Michelle Hecker, Irma Lengu, Andrea Son

## Abstract

**Background:** The American Urologic Association’s 2019 Best Practices Statement highlights the importance of procedural and host factors in optimizing antimicrobial prophylaxis for urologic procedures. For ureteral stent removal, a procedure considered low risk, the recommendation for prophylaxis is uncertain and is dependent primarily on patient factors. We examined periprocedural practices and outcomes for both low-risk and intermediate- to high-risk patients undergoing this procedure in a county hospital. **Methods:** A retrospective cohort study was performed on all patients who underwent stent removal from January to December 2019. Patients were classified as being low risk if they met the following criteria: age 48 hours within the previous 30 days, absence of external urinary catheters, no intermittent catheterization, absence of prosthetic cardiac valves, not pregnant, and not immunocompromised. All other patients were classified as intermediate to high risk. We assessed periprocedural urine testing, antimicrobial prophylaxis, and clinical outcomes. **Results:** Of 158 unique patients, 84 (53%) were classified as low risk. As shown in Table 1, preprocedural urine cultures were performed in 55% of low-risk versus 69% of intermediate- to high-risk patients. For the patients for whom urine cultures were performed, cultures were positive in 22% of low-risk versus 55% of intermediate- to high-risk patients (p < .0001). All patients received antimicrobial prophylaxis, most often a single dose after the procedure. None of the low risk patients had a positive urine culture or hospitalization within 30 days post procedure. **Conclusions:** Overall, 53% of patients undergoing stent removal were considered low-risk hosts, yet 100% of patients received antimicrobial prophylaxis. Future studies are needed to evaluate interventions to reduce unnecessary antimicrobial prophylaxis and standardize preprocedural testing in low-risk patients undergoing stent removal.

**Funding:** No

**Disclosures:** None

**Table 1.**